# Positive predictive value estimates for noninvasive prenatal testing from data of a prenatal diagnosis laboratory and literature review

**DOI:** 10.1186/s13039-022-00607-z

**Published:** 2022-07-06

**Authors:** Siping Liu, Fang Yang, Qingxian Chang, Bei Jia, Yushuang Xu, Ruifeng Wu, Liyan Li, Weishan Chen, Ailan Yin, Fodi Huang, Suxin Feng, Fenxia Li

**Affiliations:** 1 Department of Obstetrics and Gynecology, Nanfang Hospital, Southern Medical University, Guangzhou Road No.1838, 510515 Guangzhou, China; 2 Berry Genomics Corporation, Beijing, China

**Keywords:** Noninvasive prenatal testing, Positive predictive value, Chromosomal abnormalities

## Abstract

**Objective:**

Since 2011, noninvasive prenatal testing (NIPT) has undergone rapid expansion, with both utilization and coverage. However, conclusive data regarding the clinical validity and utility of this testing tool are lacking. Thus, there is a continued need to educate clinicians and patients about the current benefits and limitations in order to inform pre- and post-test counseling, pre/perinatal decision making, and medical risk assessment/management.

**Methods:**

This retrospective study included women referred for invasive prenatal diagnosis to confirm positive NIPT results between January 2017 and December 2020. Prenatal diagnosis testing, including karyotyping, chromosomal microarray analysis (CMA) were performed. Positive predictive values (PPVs) were calculated.

**Results:**

In total, 468 women were recruited. The PPVs for trisomies 21, 18, and 13 were 86.1%, 57.8%, and 25.0%, respectively. The PPVs for rare chromosomal abnormalities (RCAs) and copy number variants (CNVs) were 17.0% and 40.4%, respectively. The detection of sex chromosomal aneuploidies (SCAs) had a PPV of 20% for monosomy X, 23.5% for 47,XXX, 68.8% for 47,XXY, and 62.5% for 47,XYY. The high-risk groups had a significant increase in the number of true positive cases compared to the low- and moderate-risk groups.

**Conclusions:**

T13, monosomy X, and RCA were associated with lower PPVs. The improvement of cell-free fetal DNA screening technology and continued monitoring of its performance are important.

## Introduction

In 1997, cell-free fetal DNA (cffDNA) was first detected in the plasma of pregnant women. In 2008, massively parallel sequencing (MPS) was introduced as a new approach to non-invasive prenatal testing (NIPT) for fetal chromosomal aneuploidies [1, 2]. Several studies have shown that the NIPT has superior sensitivity and specificity to maternal serum screening for detecting trisomy 21 (T21, Down’s syndrome), trisomy 18 (T18, Edward’s syndrome), and trisomy 13 (T13, Patau syndrome) [3–5]. Over the last few years, NIPT has become the most common and first-choice screening test for fetal chromosomal abnormalities. Millions of pregnant women have undergone NIPT to identify the potential presence of T21, T18, and T13 in their fetuses, particularly in China [6–10]. NIPT has expanded to include sex chromosomal aneuploidies (SCAs), rare chromosomal aneuploidies (RCAs), and copy number variants (CNVs), although the specificity for detecting SCAs is low, and the accuracy rate for detecting RCAs/CNVs has not been well validated [9–11].

NIPT is not a diagnostic test because it measures a mixture of fetal and maternal DNA, and there is a chance of a false-positive or false-negative result, particularly for SCAs, RCAs, and CNVs. A positive result should always be confirmed with an invasive diagnostic test using amniotic fluid, chorionic villi, or umbilical cord blood. As NIPT becomes the first-tier screening test for fetal trisomies 21, 18, and 13 and other chromosomal aberrations, more studies of its validity are needed to guide clinicians regarding when to recommend testing and how to interpret the results. In the current study, we retrospectively examined 468 confirmatory diagnostic studies performed on patients who had received abnormal high-risk NIPT results and reviewed the related literatures.

## Materials and methods

### Participant recruitment

We retrospectively collected data from patients who underwent prenatal diagnosis at our laboratory owing to positive NIPT results. Data were collected between January 2017 and December 2020. Some initial NIPT was conducted at our laboratory, where as in other cases, it was performed by a variety of commercial laboratories, including BGI or Darui, or referred from other hospitals, according to their specific methodologies. For cases of NIPT performed in our laboratory, all DNA libraries were constructed from maternal plasma and subjected to MPS on the Nextseq500 (CN500) platform. Sequence reads were aligned to the human genomic sequence hg19 [12], and uniquely mapped reads were counted and normalized for GC content [6].The test sample data were finally compared to reference sample data, and Z-scores were calculated to determine chromosomal ploidy using the normal range, − 3.0 < Z < 3.0 [2]. Pregnant women with positive NIPT results were advised to undergo invasive prenatal diagnostic procedures (e.g., chorionic villus sampling [CVS]) at 10–15 weeks, amniocentesis at 16–24 weeks, or cordocentesis when the gestational age was beyond 24 weeks.

## Prenatal diagnosis

All the enrolled participants underwent an invasive diagnosis at our laboratory. The accuracy of NIPT was confirmed by karyotype analysis and chromosomal microarray analysis (CMA), which were performed in all cases with NIPT showing suspected CNV abnormalities. Karyotyping was carried out according to the conventional G-banding method. CMA was performed using a commercial SNP array chip, the Human omni-zhonghua-8 BeadChip (Illumina, Inc., San Diego, CA, USA). Aneuploid cases categorized as true positives (TPs) included those with complete concordance as well as those with partial concordance (i.e., NIPT positive for T18, confirmatory testing demonstrating trisomy for a portion of chromosome 18), those having a micro-duplication on the same chromosome by CMA, and those having incomplete concordance (i.e., NIPT positive for both T13 and T18, confirmatory testing demonstrated byT13 only). False-positive (FP)cases were defined as those with complete discordance between NIPT and confirmatory testing (i.e., NIPT positive for chr8 but with a normal karyotype and CMA demonstrating 6q27 micro-deletion).The positive predictive value (PPV) was calculated as the number of cases for which NIPT and confirmatory diagnostic testing were concordant (TP cases) divided by the number of cases with positive NIPT results (TP + FP cases) multiplied by 100.

## Data analysis and statistics

Advanced maternal age (AMA), abnormal fetal ultrasound, and adverse pregnancy history contribute to a high risk of chromosomal aneuploidies. In this study, we classified these NIPT-positive cases into three subgroups according to whether the risk of fetal abnormality was low, moderate, or high. High risk was defined as AMA, a high risk of serum screening, structural abnormality on fetal ultrasound, or a history of previous abnormal fetal pregnancy. Moderate risk was defined as the presence of ultrasound soft markers or critical risk based on serum screening. Low risk was defined as none of these known high-risk factors. The high-risk group was subdivided into two groups with one factor and two more factors. Maternal age plays an important role in fetal abnormalities, and we also classified women into AMA (≥ 35 years) and younger women. All data analyses were performed using Microsoft Excel 2007 (Microsoft Corp., Redmond, WA, USA) and SPSS version 20 (IBM Corp., Armonk, NY, USA). Differences in proportions were tested for statistical significance using the chi-square test, and a P-value < 0.05 was considered significant.

To conduct a literature review on PPVs of NIPT, a non-systematic targeted search was performed using PubMed/Medline. The keywords used were [NIPT], [non-invasive prenatal testing], [NIPS], and [non-invasive prenatal screening]. Relevant articles with more than 30,000 samples from China and 10,000 from other countries were reviewed. The search for articles progressed as the study was being completed, including additional studies identified by manually searching the references of the included articles.

## Results

### Characteristics of subjects

A total of 468 women with positive NIPT results received a prenatal diagnosis at the laboratory from January 2017 to December 2020, including 444 cases of amniocentesis (94.9%), 18 cases of cordocentesis (3.8%), and 6 cases of CVS (1.3%). These pregnant women were 17–47 years old, and the mean age was 31.7 ± 6.0 years (median 31). A total of 171 women (36.5%) were of advanced maternal age (AMA), 70 (15.0%) had maternal serum screening showing high-risk results, 146(31.2%) had ultrasound soft markers, and 59 (12.6%) had ultrasound structural abnormalities. Table 1 shows the demographic characteristics of the patients. The serum screening cut-offs for high-risk for T21 and T18 were 1/270 and 1/350, respectively, and the critical risk values were 1/271-1/1000 and 1/351-1/1000, respectively.

**Table 1:** Demographics characteristics of the 468 women under prenatal diagnosis with NIPT positive results in the laboratory

Characteristics	Cases	Constituent ratio
Ethnicity (Chinese)	468	100.0
Singleton pregnancy	450	96.2
Pregnancy		
History 1	121	25.9
History 2	137	29.3
History 3	101	21.6
History ≥ 4	109	23.3
Parity nullipara	182	38.9
Primipara	221	47.2
Mutipara	65	13.9
Results of serum screening		
High risk	71	15.2
Moderate risk	52	11.1
Low risk	56	12.0
Not performed	289	61.8
Advanced age women (AA ≥ 35)	171	36.5
Ultrasound soft indications	146	31.2
Ultrasonic structural abnormality	59	12.6
Adverse pregnancy history	28	6.0
Twin	18	3.8
IVF-ET pregnancy	21	4.5

## Cases of suspected aneuploidy

Of the 468 cases, 217 (46.4%) were common autosomal trisomies 146 (31.2%), 53 (11.3%), and 52 (11.1%) cases of T21, T18, and T13, respectively. A total of 203 TP and 166 FP cases were confirmed through invasive diagnosis. The PPVs for T21, T18, and T13 were 86.1, 57.8, and 25.0%, respectively. For the SCAs, monosomy X had the most NIPT-positive cases, while the lowest PPV was 20.0%. The PPV of XXY was the highest (68.8%), followed by that of XYY (62.5%) (Table 2).

**Table 2:** Cases of TP FP distribution in different types of aneuploides

NIPT positive result	Cases n (%)	True positive	False positive	PPV (%)	FPR (%)
T21	144	124	20	86.1	13.9
T18	45	26	19	57.8	42.2
T13	28	7	21	25.0	75.0
SCAs	146	60	86	41.1	58.9
Monosomy X	40	8	32	20.0	80.0
XXX	17	4	13	23.5	76.5
XXY	16	11	5	68.8	25.0
XYY	8	5	3	62.5	37.5
RCAs	53	9	44	17.0	83.0
CNVs	52	21	31	40.4	59.6
Total	468	247	221	52.8	47.2

Our study also included 53 patients with positive NIPT results for less common aneuploidies (i.e., monosomies 13 and 18 and trisomies for chromosomes 3, 7, 8, and 16). NIPT-positive cases of RCAs involved all rare chromosomes except chromosome 15 and chromosome 17, whereas chromosome 7 (10 cases) and chromosome 8 (8 cases) were involved in most RCA cases, followed by chromosome 16 (7 cases) and chromosome 3 (6 cases). In total, for RCAs, which exhibited the lowest PPV (17.0%), 9 TPs in 53cases were confirmed. These TP cases included one case of trisomy 7 (mosaic), one case of trisomy 9 (mosaic), one case of trisomy 12 (mosaic), and other cases with CMA results showing duplication or deletion on related chromosomes.

## Cases of suspected chromosomal deletion/duplication

Among the 52 NIPT-positive CNVs, 21 TP CNV cases were identified, and the PPV of CNVs was 40.4%.The most frequent micro-deletions in our data set were the Prader-Willi syndrome/Angelman syndrome region (PWS/AS, 15q11.2-q13.1), DiGeorge syndrome deletion (DGS, 22q11.2), Cri-du-chat syndrome region (CDC, 5pter), and del 1p36. Of the 11 cases with positive NIPT results for these recurrent regions, only one case (CDC) was confirmed. The 21 validated deletions/duplications are shown in Table 3. Among the 21 validated CNV cases, 6 CNVs were larger than 10 Mb, 4 CNVs were between 5 and 10 Mb, and 11 were smaller than 5 Mb in size. Here, we evaluated the PPV for detecting chromosomal deletions/duplications at a size of > 10 Mb, between 5 and 10 Mb, and < 5 Mb as 37.5%, 44.4%, and 44.0%, respectively.

**Table 3:** Cases with NIPT positive results for deletions/duplications validated by CMA or CNV-Seq analysis

NIPT positive result	Cases (n)	TP	FP	CMA or CNV-Seq result of TP case	Classification
CDC(5pter)	3	1	2	5p15.33p14.1(38,139 − 27,103,958)×1	Pathogenic
PWS/AS (15q11q13)	3	0	3	–	Pathogenic
DGS(22q11.2)	3	0	3	–	Pathogenic
del 1p36	2	0	2	–	Pathogenic
1q21.1q21.2 gain	2	1	1	1q21.1q21.2(144,934,509 − 147,826,275)×3	Pathogenic
2p16.3p22.2 gain	1	1	0	2p16.3p22.2(37,260,436 − 48,587,741)×3	Pathogenic
2p16.2p16.3 gain	2	1	1	2p16.2p16.3(51,091,855 − 53,835,161)×3	Uncertain significance
2p13.2p11.2 loss	1	1	0	2p11.2p13.2(72,464,241 − 88,639,638)×1	Pathogenic
3q11.2q13.11 gain (mat)	1	1	0	3q11.1q13.11(93,529,084–105,423,648)×3 (mat)	Uncertain significance
3p26.3-p26.1loss and 6p25.3-p22.1 gain	1	1	0	3p26.1p26.3(61,495-8,281,680)×1	Pathogenic
				6p25.3p22.1(137,177,381 − 155,233,098)×3	Pathogenic
4q12-13.1 gain	1	1	0	4q12q13.1(58,189,484 − 62,742,001)×3 (mat)	Uncertain significance
5p15.2p15.33 gain	1	1	0	5p15.33p15.2(38,139 − 11,343,384)×3	likely pathogenic
6p12.3 gain	1	1	0	6p12.3(43,870,083 − 50,134,803))×3	Uncertain significance
6q27 loss	1	1	0	6q27(165,218,392 − 167,604,257)×1	Uncertain significance
8p23.2 gain (mat)	1	1	0	8p23.2(3,694,817-5,950,104)×3	benign
9p24.3-p24.1 gain	1	1	0	9p24.3(46,587–822,594)×1	Uncertain significance
				9q21.11q34.3(70,693,093–141,127,261)×2 ~ 3	likely pathogenic
				17q24.2q25.3(65,066,879 − 81,051,007)×3	likely pathogenic
10q11.22q11.23 gain	1	1	0	10q11.22q11.23(47,543,535 − 51,832,220)×3	Uncertain significance
11p14.3 loss	1	1	0	11p14.3(22,937,497 − 25,859,783)×1	Uncertain significance
11q13.3q13.4 loss	1	1	0	11q13.3q13.4(70,241,935 − 72,684,898)×1	Likely pathogenic
16p13.12p12.3 loss	1	1	0	16p13.11p12.3(15,479,879 − 18,164,692)×1	Uncertain significance
16q12.3q13.11 gain	3	1	2	4q35.1(185,776,067–187,061,084)×1;	likely pathogenic
				16p13.11p12.3(15,132,108 − 18,801,583)×3	Uncertain significance
16q23.1q23.2 loss	1	1	0	16q23.1q23.2(77,237,983 − 79,710,481)×1	Uncertain significance
18q21.3q22.3 loss	1	1	0	seq[hg19]del(18)(q21.31-q22.3); chr18(g.54,900,000–69,940,000)	Pathogenic
20p13p12.1 loss	1	1	0	20p13p12.2(2,648,482 − 11,807,011)×1	Pathogenic
22q11.21 loss	3	0	3	–	–

## NIPT accuracy with other risk factors and serum screening

P-values were calculated to compare TP and FP case distributions among the different risk groups (Table 4). The number of TP cases increased significantly in the two high-risk groups, comprising 54.0% and 87.5%, respectively, whereas these values were only 32.0% and 39.6% in the low- and moderate-risk groups, respectively. Significant differences between the low-and high-risk groups were found (P-values < 0.001), but no significant difference was observed between the low- and moderate-risk groups (P = 0.276). T21/T18/T13 showed similar TP and FP case distributions, and TP cases comprised 72.5% and 97.1% of the high-risk groups with one and two or more factors, respectively, compared to the low-risk group (36.4%) and moderate-risk groups (57.1%), both of which were significantly different (P < 0.001). No significant difference was observed between the low- and moderate-risk groups (P = 0.086) (Table 4).

**Table 4:** Cases of TP and FP distributions in different risk groups

Group	Total	True Positive	False Positive	PPV (%)	*P* value
All aneuploides					
Low risk	100	32	68	32.0	
Moderate risk	91	36	55	39.6	0.276
High risk-one factor	189	102	87	54.0	< 0.001
High risk-two factors or more	88	77	11	87.5	< 0.001
T21/T18/T13					
Low risk	33	12	21	36.4	
Moderate risk	35	20	15	57.1	0.086
High risk-one factor	80	58	22	72.5	< 0.001
High risk-two factors or more	69	67	2	97.1	< 0.001

Of the 171 AMA cases, there were 111 TP cases (PPV, 64.9%) and 60 FP cases, while in the group of women < 35 years, 136 TP (PPV, 45.8%) and 161 FP cases were confirmed (P < 0.001). Significant increases in TP% were also observed in the AMA group compared to the younger female group for T21/T18/T13 (P < 0.001).

Of the 468 cases, 179 cases (38.2%) had a serum screening result, including 71 cases of high risk, 52 cases of moderate risk, and 56 cases of low risk. The PPVs were 70.4% in high risk serum screening result group, 48.1% in moderate risk serum screening, and 48.2% in low risk serum screening result. Significant differences between the low-and high-serum screening risk groups were found (P-values = 0.011).

## Discussion

In this study, we presented the PPVs for NIPT of different types of aneuploidies, including T21, T18, T13, SCAs, RCAs, and CNVs. Here, we summarized previously reported literature on PPVs for NIPT, using data collected from 2015 (Table 5). These data were mostly from China [8–10, 13–20] and the United States [23, 27], the Netherlands [24], Japan [22], Iran [25], and Italy [21, 26]. The PPVs presented in this study were consistent with those in previous reports (Table 5) [9, 15–17, 19, 20]. PPVs for T21 were higher than 84% in most reports, while only two reports had PPVs lower than 80% [10, 14]. These two reports also showed lower PPVs for other aneuploidies. After reviewing the literature, we found that they both conducted sequencing using the JingXin Bioelectron Seq 4000 System (CFDA registration permit No. 20153400309). The lower PPVs may be caused by the sequence-read depths and algorithms provided by the system. Most reports from China show PPVs of T21 ranging from 80 to 92%, which is consistent with our study [8, 9, 18–26]. Reports from outside China also showed higher PPVs for T21, ranging from 95.7 to 99.2% [21–27]. These reports from China showed higher PPVs on T18, T13, and SCAs, which we believe was caused by the participant women having a higher risk for aneuploidies; the mean age was 35.3 years in a report from Italy [21], and the average maternal age was 34 years in a report from the USA [27], while the mean age was 31 years in this study. The PPVs of T18 ranged from 45 to 82% in most reports, except for reports of high-risk populations with higher PPVs [21–27]. For T13, the PPVs were lower than 50% in studies based on the Chinese population and some similar reports [8–10, 14, 15, 18–20]. The PPVs for RCAs were lower than 28%, which may be due to the low prevalence of the disorder, except for in one study with a high-risk population [21]. For CNVs, the PPVs ranged from 29 to 50%, except in one study with a high-risk population [27]. Differences in PPVs between studies occur because study populations differ in size, demographics, and clinical characteristics, and different NIPT platforms with variable sequence read depths and algorithms are used by different providers. We also summarized the PPVs of SCAs in Tables 6 and 45X had the lowest PPVs, ranging from 14 to 29% in the general population [9, 13, 16, 28, 29]. The PPVs of XXX, XXY, and XYY varied greatly, which we believe was due to the small number of sex aneuploidies in each study. PPV is a population-based figure that reports the chance that a positive NIPT result is reflective of the karyotype of the fetus, and PPVs are involved with the prevalence of a disorder [31]. Thus, it is vital to educate ordering physicians regarding the differences between specificity and PPV. Sensitivity indicates the chance that a test result will be positive for a given disorder; specificity measures the chance that a fetus, which does not have a particular aneuploidy, will test negative for that aneuploidy. Neither sensitivity nor specificity reflected the prevalence of a disorder in the population; however, PPV did. For an average clinician, the claim that a test is > 99% specific leads him or her to expect a false-positive rate of < 1%. As we can see in the study and other previous reports, the ability of NIPT to correctly predict a positive result for T18 is less than 80% and less than 50% for T13, monosomy X, and RCAs. The study revealed that a significant increase in PPVs was found in high-risk groups with one factor and two or more factors, which also indicates that women with AMA, along with other high-risk factors, such as having a high-risk serum screening result, can undergo invasive diagnosis and not other screening. While in the study, before taking NIPT, there were 179 cases (38.2%) having a serum screening result, including 71 cases of high risk.

**Table 5:** Summary of the findings of PPVs in different types of aneuploides

Reports	Country	NIPT cases	NIPT positive cases/Validated(%)	PPV					
				T21	T18	T13	SCAs	RCAs	CNVs
Zhang [8]	China	146,958	1578/1055 (66.9)	92.2	76.6	32.8	–	–	–
Xue [9]	China	57,204	856/671 (78.4)	80.4	65.3	19.5	26.8	–	–
Chen [10]	China	42,910	534/403 (75.5)	79.2	54.8	13.8	33.0	9.4	29.0
Liang [13]	China	94,085	965/965 (100)	94.5	82.1	46.2	46.7	28.6	40.8
Liu [14]	China	42,924	281/281 (100)	78.5	63.0	10.0	47.2	–	–
Luo [15]	China	40,311	468/398 (85.0)	84	48.2	14.3	34.9	9.3	–
Xu [16]	China	31,515	434/307 (70.7)	84.1	69.4	46.7	42.7	–	–
Lu [17]	China	36,913	371/277 (61.2)	84.7	58.7	41.9	33.3	–	–
Xu [18]	China	44,578	773/374 (48.4)	96.0	75.5	20.0	–	–	–
Wang [19]	China	39,002	473/338(71.5)	88.9	53.3	20	40.2	7.3	49.0
Shi [20]	China	36,970	237/171(72.2)	81.3	47.6	17.6	56.5	–	50.0
Fiorentino [21]	Italy	12,078	196/169 (86.2)	98.9	93.8	92.3	75.0	58.8	–
Samura [22]	Japan	30,613	554/462 (83.4)	96.5	82.8	63.6	–	–	–
Guy [23]	USA	69,794	1359/478 (35.2)	98.1	88.2	59.3	69.0	–	–
van der Meij [24]	Netherlands	73,239	343/303 (88.3)	96	98	53	–	6	–
Garshasbi [25]	Iran	11,223	180/170 (94.4)	95.7	92.3	87.5	70.0	–	–
La Verde [26]	Italy	36,456	489/472 (96.5)	99.2	91.2	84.4	86.7	–	–
Soster [27]	USA	53,099	2687/1569 (58.4)	96.3	94.2	76.0	–	22.4	72.6

**Table 6:** Summary of the findings of PPVs in SCAs

Reports	Country	NIPT cases	NIPT positive SCAs cases/Validated	PPV			
				45X	XXX	XXY	XYY
Xue [9]	China	57,204	295/NA	19.4	55.6	55.2	85.7
Liang [13]	China	94,085	390/390 (100)	25.8	61.7	82.9	75
Xu [28]	China	32,931	140/101 (72.1)	26.1	85	85	68.8
Deng [29]	China	50,301	308/182 (59.1)	18.4	44.4	39.3	75
Xu [16]	China	31,515	225/143 (63.6)	26	65.2	75	83.3
Fiorentino [21]	Italy	12,078	48/36 (75.0)	58.3	93.3	85.7	100
Garshasbi [25]	Iran	11,223	29/29 (100)	66.7	66.7	80	100
La Verde [26]	Italy	36,456	145/135 (93.1)	85.2	83.3	87.5	100
Soster [27]	USA	53,099	556/193 (34.7)	55.3	70.8	95.8	100
Lüthgens [30]	Germany	66,203	351/144 (41.0)	29	29.7	57.5	80

The American College of Obstetricians and Gynecologists (ACOG) published the clinical management guidelines for screening for fetal chromosomal abnormalities in 2020, stated that patients should have one prenatal screening approach and should not have multiple screening tests performed simultaneously [31]. Another study also reported that NIPT should not be recommended for pregnancies with ultrasound anomalies or high-risk pregnancies, even the expanded NIPT [32, 33]. Nonetheless, some pregnant women consider NIPT an acceptable alternative to invasive diagnostic testing. For post counseling, in regard to current NIPT guidelines, the ACMG strongly suggests invasive prenatal testing to confirm all positive findings [34, 35].

## Conclusions

In conclusion, we evaluated and summarized the PPVs of all aneuploidies using NIPT in this study and in previous publications. The NIPT is fundamentally a screening test and cannot be used as a replacement for invasive prenatal diagnosis. Based on the study findings, we hope to raise concerns about the limitations of NIPT. Furthermore, we have to ensure that clinicians interpret the NIPT results correctly and provide more careful and precise counsel so that pregnant women can make a more informed decision based on scientific data and knowledge.

## Data Availability

The data that support the findings of this study are available from the corresponding author upon reasonable request.

## Abbreviations

NIPT: Noninvasive prenatal testing
CMA: Chromosomal microarray analysis
PPV: Positive predictive value
cffDNA: Cell-free fetal DNA
SCA: Sex chromosomal aneuploidies
RCA: Rare chromosomal aneuploidies
CNV: Copy number variants
CVS: Chorionic villus sampling
